# Urinary TMAO Levels Are Associated with the Taxonomic Composition of the Gut Microbiota and with the Choline TMA-Lyase Gene (*cutC*) Harbored by Enterobacteriaceae

**DOI:** 10.3390/nu12010062

**Published:** 2019-12-25

**Authors:** Alessandro Dalla Via, Giorgio Gargari, Valentina Taverniti, Greta Rondini, Ilaria Velardi, Veniero Gambaro, Giacomo Luca Visconti, Valerio De Vitis, Claudio Gardana, Enzio Ragg, Andrea Pinto, Patrizia Riso, Simone Guglielmetti

**Affiliations:** 1 Department of Food, Environmental and Nutritional Sciences (DeFENS), University of Milan, 20122 Milan, Italy; alessandro.dallavia@unimi.it (A.D.V.); gargari.g@gmail.com (G.G.); valentina.taverniti@unimi.it (V.T.); gremary8687@gmail.com (G.R.); i.velardi@hotmail.com (I.V.); valeriodevitis88@gmail.com (V.D.V.); claudio.gardana@unimi.it (C.G.); enzio.ragg@unimi.it (E.R.); andrea.pinto@unimi.it (A.P.); patrizia.riso@unimi.it (P.R.); 2 Department of Pharmaceutical Sciences, University of Milan, 20122 Milan, Italy; veniero.gambaro@unimi.it (V.G.); giacomo.visconti@unimi.it (G.L.V.)

**Keywords:** choline, trimethylamine, trimethylamine n-oxide, 16S rRNA gene profiling, qPCR, linear mixed models

## Abstract

Gut microbiota metabolization of dietary choline may promote atherosclerosis through trimethylamine (TMA), which is rapidly absorbed and converted in the liver to proatherogenic trimethylamine-N-oxide (TMAO). The aim of this study was to verify whether TMAO urinary levels may be associated with the fecal relative abundance of specific bacterial taxa and the bacterial choline TMA-lyase gene *cutC*. The analysis of sequences available in GenBank grouped the *cutC* gene into two main clusters, cut-Dd and cut-Kp. A quantitative real-time polymerase chain reaction (qPCR) protocol was developed to quantify *cutC* and was used with DNA isolated from three fecal samples collected weekly over the course of three consecutive weeks from 16 healthy adults. The same DNA was used for 16S rRNA gene profiling. Concomitantly, urine was used to quantify TMAO by ultra-performance liquid chromatography coupled with tandem mass spectrometry (UPLC-MS/MS). All samples were positive for *cutC* and TMAO. Correlation analysis showed that the cut-Kp gene cluster was significantly associated with *Enterobacteriaceae*. Linear mixed models revealed that urinary TMAO levels may be predicted by fecal cut-Kp and by 23 operational taxonomic units (OTUs). Most of the OTUs significantly associated with TMAO were also significantly associated with cut-Kp, confirming the possible relationship between these two factors. In conclusion, this preliminary method-development study suggests the existence of a relationship between TMAO excreted in urine, specific fecal bacterial OTUs, and a *cutC* subgroup ascribable to the choline-TMA conversion enzymes of *Enterobacteriaceae*.

## 1. Introduction

From infancy, the microorganisms colonizing the human gastrointestinal tract (GIT), collectively known as GIT microbiota, act as a “hidden” metabolic organ that exerts indispensable functions for the development and physiology of the human organism, such as the production of vitamins, modulation of the immune system, competitive exclusion toward exogenous pathogenic bacteria, xenobiotic detoxification, and production of short-chain fatty acids [1]. Nonetheless, detrimental activities have also been associated with gut commensal microorganisms, such as the production of carcinogens by the bacterial nitroreductases and azoreductases [2], or the conversion of primary bile acids to toxic compounds by the microbiota-associated enzyme cholesterol dehydrogenase and 7-α-dehydroxylase [3]. In addition, it was proposed that the intestinal bacterial enzymatic activities that produce trimethylamine (TMA) may promote atherosclerosis. TMA, in fact, is readily absorbed from the intestinal tract and, once in the liver, is converted into trimethylamine-N-oxide (TMAO) [4], whose plasma level has been identified as a metabolite strongly associated with atherosclerosis in a large case-control cohort for cardiovascular disease [5]. In particular, TMAO was proposed to promote atherogenesis by increasing cholesterol in macrophages and enhancing the accumulation of foam cells in artery walls [4,5]. Nonetheless, the literature has contradicted the role of TMAO, and recent studies have questioned its deleterious role in the cardiovascular system [6], suggesting, on the contrary, that TMAO could have protective functions [7,8].

Reportedly, a dominant contribution to the production of TMA in the gut comes from the microbial metabolism of diet-derived substrates such as carnitine- and choline-containing molecules [4,5,9]. Choline is an essential nutrient that is used by cells to synthesize membrane phospholipids. Furthermore, choline is the precursor of the neurotransmitter acetylcholine and a major source for methyl groups via its metabolite, trimethylglycine (betaine) [10]. The main dietary sources of the choline moiety, which is mostly present in food as lecithin (i.e., phosphatidylcholine), were reported to be eggs, liver, soybeans, and pork [11]. Although they are also present in numerous other foods [12], recent surveys in the USA indicated that choline may be underconsumed in specific populations (e.g., pregnant women and vegans) [13]. Based on the average observed choline intake in healthy European populations, a panel of the European Food Safety Authority set the adequate intake of choline at 400 mg/day [14].

Recent literature has suggested that the enhanced abundance of choline utilization genes in the intestinal microbiome is associated with increased TMA levels in the gut and, subsequently, with a higher hepatic production of TMAO. Proof of the importance of choline-derived TMA in the context of TMAO toxicity was recently provided by the study of Craciun and Balskus, in which the specific inhibition in mouse intestine of the microbial choline TMA-lyase (the primary enzymatic activity involved in the production of TMA from choline [15]) resulted in a significant reduction in plasma TMAO levels and recovery from dietary-induced platelet aggregation and thrombus formation [16].

Choline TMA-lyase is discontinuously distributed in bacterial taxa. Consequently, it was speculated that the phylogenetic composition of the microbiota is plausibly a poor predictor of the intestinal potential to convert choline into TMA [15,17,18]. However, in another study, the taxonomic structure of the gut microbiota was used to predict genes involved in choline metabolism [19] by means of PICRUSt, a bioinformatic tool used to infer the functional profiles of the microbial communities from 16S rRNA gene profiling data [20]. Although the toxicity of TMAO has been extensively investigated in the last 10 years, the association potentially existing among host TMAO levels, gut microbiota composition, and the intestinal microbial metabolization of choline has been only marginally considered. In this context, we developed a molecular protocol for the targeted quantification in the fecal microbiome of the bacterial gene *cutC* coding for the glycyl radical enzyme homolog choline TMA-lyase [15,21]. This protocol was applied to quantify the *cutC* gene abundance in the fecal samples collected at different time points from a group of healthy adults. Then, the obtained results were analyzed in comparison with the bacterial taxonomic composition and the urinary levels of TMAO concomitantly determined in the same population to deduce the potential association of excreted TMAO with gut microbial taxa and/or specific choline TMA-lyase enzymes.

## 2. Materials and Methods

### 2.1. Design and Use of Primers Targeting the *cutC* Gene

The primers used in polymerase chain reaction (PCR) for the amplification of the *cutC* gene were designed as follows. The GenBank database and Conserved Domain Database (CDD) at the National Center for Biotechnology Information (NCBI) were queried to select 52 nonredundant representative bacterial proteins of the choline trimethylamine-lyase protein family TIGR04394 (choline_CutC; EC Number 4.3.99.4), including the CutC enzymes of *Desulfovibrio desulfuricans* [4], and *Klebsiella pneumoniae* [22]. Then, the corresponding CDS nucleotide sequences of selected proteins were used to build a UPMGA tree upon ClustalW multiple alignments. According to the obtained dendrogram, sequences were clustered in two groups: One including the *cutC* sequence of *K. pneumoniae*, named cut-Kp, and one including the *cutC* sequence of *D. desulfuricans*, named cut-Dd (Supplementary Figure S1). Finally, a pair of primers was designed in the most conserved regions of each group of sequences: cut-Dd-F, 5′-CGTGTTGACCAGTACATGTA-3′ and cut-Dd-R 5′-GCTGGTAACCTGCGAAGAA-3′ (expected amplicon of 185 bp); cut-Kp-F, 5′-GATCTGACCTATCTGATTATGG-3′, and cut-Kp-R, 5′-TTGTGGAGCATCATCTTGAT-3′ (expected amplicon of 190 bp).

### 2.2. PCR Detection of *cutC* Gene in Single Strains

The two primer pairs designed as described above were used in endpoint PCR with the genomic DNA extracted from 64 bacterial strains (Table S1). Reaction mix was prepared in 25 μL, including 0.5 units of DreamTaq Polymerase (ThermoFisher, Fermentas, Waltham, MA, USA), 1× concentration of DreamTaq Polymerase Buffer (ThermoFisher, Fermentas,), 0.25 μM of each primer, 200 μM of deoxyribonucleotide triphosphate (dNTPs), and 0.5 mM of MgCl2. The PCR cycle program used was the following: Initial denaturation at 95 °C for 2 min, followed by 35 cycles of denaturation at 94 °C for 45 s, annealing at 58 °C for 45 s for the cut-Dd couple and 56 °C for 45 s for the cut-Kp couple, and extension at 72 °C for 20 s. A final extension of 7 min at 72 °C was then applied.

### 2.3. Detection of Choline-Utilization Activity in Single Strains

Bacterial strains were grown in the respective culture medium (reported in Table S1) for 48 h. Afterward, the biomasses were collected by centrifugation at 9500 g for 10 min. The cell pellets were then washed with sterile PBS and resuspended in fresh medium with the addition of 0.2% filter-sterilized choline. Bacteria were incubated at 37 °C for 48 h in glass tubes with screw cap. Afterward, supernatants were collected and used for mass spectrometry (MS) and nuclear magnetic resonance (NMR) analyses. The MS analyzes were performed by directly injecting 5 μL of diluted broth cultures after the removal of the bacterial cells by centrifugation and subsequent filtration with a 0.45-μm syringe filter. In detail, the broth cultures were analyzed in full scan in the range from 50 u to 400 u on an HR-MS Orbitrap model Exactive with a HESI-II probe for electrospray ionization (Thermo Scientific, San Jose, CA, USA). The resolution, gain control, mass tolerance, and maximum ion injection time was set to 50 K, 1E6, 2 ppm, and 100 ms, respectively. The MS data were processed using Xcalibur software (Thermo Scientific). Choline and TMA were used as reference standard. Choline and TMA were also directly detected in broth cultures by ^1^H-NMR with a 60 MHz benchtop NMR spectrometer Spinsolve 60 Carbon Ultra, Magritek GmbH (Aachen, Germany).

### 2.4. Study Population

Study participants were recruited within the University campus. In total, four females and 12 males aged 21–45 (mean: 29.8 years) were enrolled (Table S2). The inclusion criteria were as follows: Healthy adult volunteers of both sexes who provided signed informed consent of their participation in the study. The exclusion criteria were as follows: Antibiotic consumption in the month preceding the start of the study, consumption of antacids or prokinetic gastrointestinal drugs, episodes of viral or bacterial enteritis in the two months prior to the study, episodes of gastric or duodenal ulcers in the previous five years, pregnancy or breastfeeding, recent history of alcohol abuse or suspected drug use, and any severe disease that may interfere with treatment. Ethical permission was granted by the University of Milan Ethics Committee (ref: opinion no. 37/16, 15 December 2016).

### 2.5. Collection of Fecal and Urine Samples 

Three fecal sample were collected weekly over the course of three consecutive weeks from each volunteer. All the participants were asked to follow their regular diet during the three weeks. Concomitantly to the fecal sample, the volunteers provided 24-h urine collection.

Urine samples were collected over 24 h in sterile tanks and on the same days that fecal samples were been collected. The volume of collected urine was recorded in order to calculate the daily excretion of trimethylamine oxide (TMAO). Immediately after delivery, part of the urine samples was transferred in 10-mL sterile tubes and stored at −80 °C until analysis.

### 2.6. Analysis of *cutC* Gene by Quantitative Real-Time PCR

The cutC gene was quantified in fecal DNA with quantitative real-time PCR (qPCR) with both primer pairs, cut-Dd and cut-Kp. To this aim, DNA was extracted from feces using the kit PowerLyzer^®^ PowerFecal^®^ DNA Isolation Kit (MO BIO Laboratories, Inc.), starting from 0.25 ± 0.02 mg of sample according to the manufacturer’s instructions. Primer pairs were tested with a gradient qPCR in a range of eight temperatures in order to find the most efficient annealing temperature using DNA of Streptococcus dysgalactiae 485 and Klebsiella sp. A1.2 as reference DNA. In addition, the amplification efficiency of the two pairs of primers was tested in qPCR experiments with six serial 1:3 dilutions of genomic DNA isolated from *Streptococcus dysgalactiae* 485, *Klebsiella* sp. A1.2, and human fecal metagenomic DNA. All DNA (bacterial and metagenomic) serial dilutions were tested with primer concentrations of 0.5 µM, 0.4 µM, and 0.3 µM. Efficiency curves were obtained with Bio-Rad software by setting samples as “standard” and obtaining a curve with efficiency (E) parameter and R2 value. Based on the results of these setup experiments, primers were then used at a final concentration of 0.5 µM, as with this concentration, we obtained an R2 value of 0.98. In addition, two randomly selected fecal DNA samples were tested at the different concentration by adding 70 ng, 50 ng, 25 ng, and 10 ng in qPCR reactions. Based on Ct value comparison between the different DNA concentrations, the *cutC* gene quantification was subsequently performed using 50 ng of total DNA. The reaction mix contained the SsoFast TM Eva-SuperGreen Supermix 2× (Bio-Rad Laboratories), deionized Milli-Q water (Millipore), and primers. All DNA samples (5 µL in each well) were tested in technical duplicate. The qPCR cycles employed were the following: Initial denaturation at 95 °C for 3 min, followed by 44 cycles of denaturation at 95 °C for 30 s, annealing at 58 °C (for cut-Dd primers) or 58.5 °C (for cut-Kp primers) for 30 s, and elongation at 72 °C for 5 s. A final denaturation ramp between 65 °C and 95 °C for 5 s was performed for the melting curve analysis. Moreover, specificity of qPCR reaction was confirmed by checking the presence of only one amplification and of the expected size in electrophoresis on a 2% agarose gel. A total of 48 fecal samples were analyzed. Each sample was analyzed with each primer set in duplicate. The 2^−ΔΔCt^ method was used for the relative quantification of *cutC* gene, using the EUB panbacterial primers [Muyzer] targeting the 16S rRNA gene as reference. Data were reported as relative increase of *cutC* copy number compared to the level of the sample that showed the highest significant Ct in qPCR set as 1.

### 2.7. Analysis of the Bacterial Taxonomic Composition of Fecal Samples

The bacterial community structure of the fecal microbiota was analyzed as described elsewhere [23,24], with DNA extracted from feces as described in Section 2.2. In brief, extracted DNA was analyzed through 16S rRNA gene profiling. Sequencing reads were generated at the Institute for Genome Sciences (University of Maryland, School of Medicine, Baltimore, MD, USA) with Illumina HiSeq 2500 rapid run sequencing of the V3–V4 variable region. Sequencing reads were equally distributed among the samples. Sequences were filtered and trimmed based on their quality. We obtained a sequence length of 301 bp for both R1 and R2 sequences with an average quality score (Phred score) higher than 35. Sequencing reads were rarefied at 5000 per sample. Subsequently, sequence reads were analyzed through the bioinformatic pipeline Quantitative Insights into Microbial Ecology (QIIME) version 1.9.1 [25] with the GreenGenes database updated to version 13.5. The relative abundance of bacteria in each fecal sample was reported at the taxonomic levels of phylum, class, order, family, genus, and operational taxonomic units (OTUs). Sequence were deposited in the European Nucleotide Archive (ENA) of the European Bioinformatics Institute under accession code PRJEB34169.

### 2.8. TMAO Quantification in Urine Samples

TMAO levels in urine samples were determined by ultra-performance liquid chromatography coupled to tandem mass spectrometry (UPLC-MS/MS) (Waters Acquity UPLC system). The analysis method involved the use of a totally porous column with stationary C8 stable bond (Agilent Poroshell C8-SB) and a mobile phase consisting of a gradient acetonitrile and formate buffer (3 mM of ammonium formate and 0.1% formic acid). The UPLC system was equipped with a triple quadrupole detector, which allowed the development of a “multiple reaction monitoring” (MRM) method for the analysis of TMAO. In detail, once thawed at room temperature and after centrifugation at 6000 rpm for 5 min, 25 µL of urine sample were diluted in 950 µL of UPLC mobile phase (1/1 (*v*/*v*) acetonitrile/ultra-pure sterile water + 0.025% of formic acid), and 25 µL of deuterated internal standard solution (1 ppm, TMAO-d9, Spectra 2000) were used for the normalization of results [26]. The UPLC samples were prepared mixing 950 µL of mobile phase [1/1 (*v*/*v*) acetonitrile/ultra-pure sterile water + 0.025% formic acid), 25 µL of urine sample, and 25 µL of deuterium-labeled methyl d9-TMAO solution (1 ppm; Spectra 2000 S.r.l., Roma, Italy). Mobile phase: 1/1 (*v*/*v*) acetonitrile/ultra-pure sterile water + 0.025% of formic acid. The run time per sample was 8 min. Sample freezing and thawing or their prolonged storage at room temperature did not have an impact on the TMAO quantification. A triple set of working standards of TMAO (trimethylamine N-oxide dihydrate, Fluka) at concentrations of 5 ppm, 50 ppm, 100 ppm was prepared according to the method described above, replacing the 25 µL of urine sample with 25 µL of standard solution. The average response factor was used for calculation.

### 2.9. Statistical Analysis

Statistical analyses of data were carried out using R statistic software (version 3.4.2). Concerning *cutC* gene and TMAO data, intrasubject variability was defined “high” when variance among the three replicates results were higher than twice the median of all variances. Correlation analyses were performed using the Kendall and Spearman formula with the items specified in the text as predictors and dependent variables. Significance was set at *p* ≤ 0.05, and mean differences in the range 0.05 < *p* < 0.10 were accepted as trends. To find associations among TMAO levels, bacterial taxa relative abundance, and *cutC* gene abundance, the machine learning supervised linear mixed model (LMM) algorithm was used. In brief, the LMM was performed using “lmer” function in the “lme4” library [27]. All samples were used in the LMM analysis (n = 48), considering that three measurements were available for each subject. The Akaike’s Information Criterion (AIC) was used to test the goodness of fit of the LMM. The AIC index/value depends on the ANOVA test results between two models: The model that considered the effect of the predictors and the null model. 

## 3. Results

### 3.1. Distribution of the *cutC* Gene among Bacterial Taxa

According to the literature, the ability of intestinal bacteria to convert the choline moiety to TMA is primarily associated with a recently discovered choline utilization (cut) genetic region harboring the *cutC* gene, which encodes a glycyl radical enzyme catalyzing C–N bond cleavage [15,18]. For this reason, we designed primers specifically targeting the *cutC* gene. These primers were intended for quantitative PCR (qPCR) experiments, and we avoided the use of degenerations in their sequence. In contrast, to target all putative *cutC* sequences identified in GenBank, we clustered the putative *cutC* genes into two groups (named Dd and Kp) according to sequence similarity (Supplementary Figure S1) and designed a pair of primers for each group in the most conserved sequence regions. Group Dd included putative *cutC* genes from *Firmicutes* (*Anaerococcus*, *Clostridium*, *Enterococcus*, *Streptococcus*), *Proteobacteria* (*Desulfotalea*, *Desulfovibrio*, *Enterobacter*), and *Actinobacteria* (*Olsenella*). Group Kp comprised putative *cutC* gene sequences from *Proteobacteria* (*Aeromonas*, *Enterobacter*, *Erwinia*, *Escherichia*, *Klebsiella*, *Pectobacterium*, *Pelobacter*, *Proteus*, *Providencia*, *Raoultella*, *Serratia*) and *Firmicutes* (*Desulfosporosinus*, *Enterococcus*).

**Figure 1:**
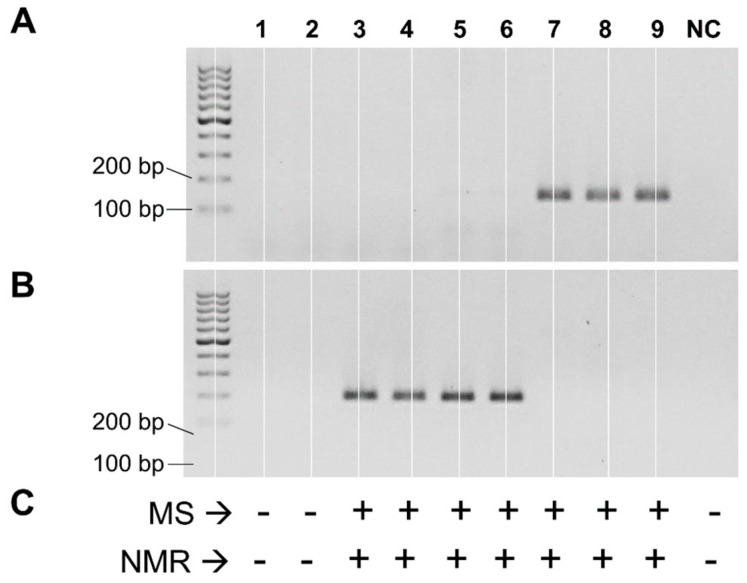
Detection of the choline-utilization activity in pure bacterial cultures. Panels (**A**,**B**) represent agarose gel resulting from end-point PCR with primers cut-Dd (**A**) and cut-Kp (**B**). Panel (**C**) summarizes the detection of TMA in cell-free broth by mass spectrometry (MS) and nuclear magnetic resonance (NMR); +, TMA detected; -, TMA not detected. Lanes: **1**, *Escherichia coli* 3.1; **2**, *Lactococcus garvieae* FMBgr; **3**, *Enterococcus gilvus* MD160; **4**, *Enterococcus hirae* MD179; **5**, *Klebsiella oxytoca* MIMgr; **6**, *Klebsiella* sp. A1.2; **7**, *Streptococcus dysgalactiae* 485; **8**, *Streptococcus dysgalactiae* 486; **9**, *Streptococcus dysgalactiae* A 1.2; NC, negative control (i.e., M17 broth incubated without bacteria).

Subsequently, the two primer sets were used in endpoint PCR reactions to test the presence of putative *cutC* genes within the genomic DNA isolated from the pure cultures of 64 bacterial strains. We obtained an amplicon of the expected size from seven strains. Specifically, strains *Streptococcus dysgalactiae* 485, *S. dysgalactiae* 486, and *S. dysgalactiae* A1.3 gave a band of the expected size with primers cut-Dd. In addition, strains *Enterococcus gilvus* MD179, *Enterococcus hirae* MD160, *Klebsiella oxytoca* MIMgr, and *Klebsiella* sp. MIMgr were positive with primers cut-Kp (Figure 1A,B). MS and NMR analyses revealed the ability to metabolize choline and produce TMA only for the same seven strains that resulted in positive PCR experiments (Figure 1C and Supplementary Figure S2).

### 3.2. Bacterial Taxonomic Structure of the Fecal Microbiota

The metagenomic DNA isolated from the feces collected at three time points from 16 healthy adults (n = 48) was used in 16S rRNA gene profiling experiments. A total of 12,588,795 filtered high-quality sequence reads were generated with an average of 13,340 ± 8677 (mean ± standard deviation; max-min 11,594–4570) per sample.

We failed to stratify samples according to the 16S rRNA gene profiling data, indicating that fecal bacterial community structure was homogeneous among samples and among subjects (Supplementary Figure S3). In addition, we also observed that the overall composition of the fecal microbiota in each subject remained mostly stable over the three collection time points (Supplementary Figure S3). Globally, 182 bacterial genera were estimated, with a minimum of 36 and a maximum of 98 genera per fecal sample. *Bacteroides* was the most prevalent genus, followed by four genera of the order *Clostridiales* (undefined *Ruminococcaceae*, undefined *Lachnospiraceae*, *Ruminococcus*, and *Faecalibacterium*) (Supplementary Figure S4A). At the family level, most of the reads were ascribed to only three families, i.e., *Ruminococcaceae*, *Bacteroidaceae*, and *Lachnospiraceae* (Supplementary Figure S4B).

### 3.3. Putative *cutC* Genes in Human Fecal Metagenomic DNA

**Figure 2:**
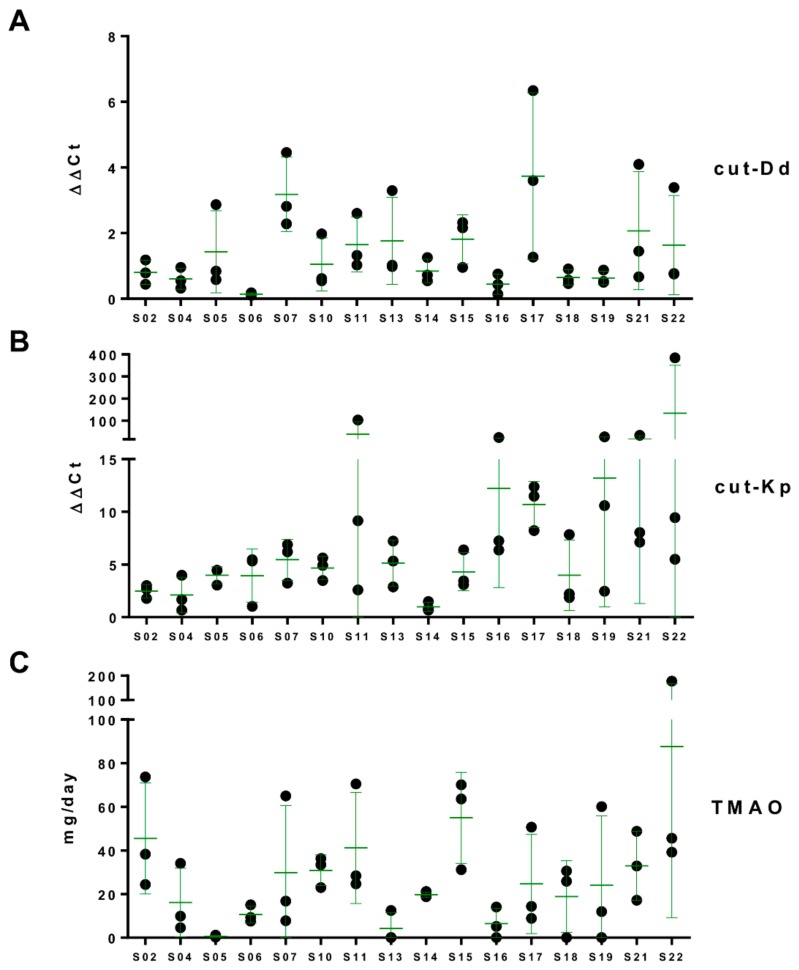
Fecal levels of the *cutC* gene and daily urinary excretion of trimethylamine-N-oxide (TMAO). The relative abundance of *cutC* was determined by quantitative real-time polymerase chain reaction (qPCR) with the primer pair cut-Dd-F/R (panel **A**) and cut-Kp-F/R (**B**). The TMAO concentration was determined by ultra-performance liquid chromatography coupled with tandem mass spectrometry (UPLC-MS/MS) in urine collected over 24 h (**C**). Green bars represent the mean ± standard deviation of three measurements per subject.

In order to investigate the presence of *cutC* genes in the human gut microbiome, the cut-Dd and cut-Kp primer sets were used in qPCR experiments using the same fecal metagenomic DNA as a template from healthy adults used for microbiota profiling. All analyzed fecal samples gave a positive signal in qPCR with both primer pairs (Figure 2). In general, cut-Kp was detected at a higher relative concentration than cut-Dd (median ΔΔCt of 5.33 and 0.85 for cut-Kp and cut-Dd, respectively) (Figure 2A,B). In addition, with both cut-Kp and cut-Dd, six volunteers out of 16 showed a variance among the three replicates that was higher than twice the median of all variances, indicating a higher intrasubject variability (Figure 2A,B).

Subsequently, we performed correlation analyses between the *cutC* abundances determined with qPCR and the 16S rRNA gene profiling data to find potential relationships between the choline TMA-lyase genes and specific bacterial taxa of the fecal microbiota. To this end, we used the median relative abundance of bacterial taxa in fecal samples as predictors, whereas the dependent variables considered were the median abundances of cut-Dd and cut-Kp determined by qPCR per subject. We found that cut-Dd was positively correlated with taxa belonging to the phylum *Firmicutes*, including an undefined *Mogibacteriaceae* genus, *Oscillospira*, and the family *Christensenellaceae*. On the contrary, cut-Dd was negatively correlated with the *Firmicutes* order *Bacillales*, the *Firmicutes* genus *Streptococcus*, and the *Proteobacteria* genus *Haemophilus* (Supplementary Figure S5). Conversely, cut-Kp was positively associated with *Proteobacteria*. In particular, inside this phylum, a significant correlation was found with the family *Enterobacteriaceae* (Supplementary Figure S5).

### 3.4. Daily Urinary Excretion of TMAO

Subjects were asked to collect 24-h urine specimens the same days when the fecal samples were taken. Then, the levels of TMAO were quantified by UPLC-MS in all urine samples, revealing wide variability among the investigated healthy adults, with levels of urinary TMAO excretion ranging from less than 1 mg to more than 175 mg per day (Figure 2). We also observed an evident intrasubject variability in five volunteers whose TMAO excretion showed a variance among the three replicates that was higher than twice the median of all variances (Figure 2C). In particular, four out of the five volunteers with wide intrasubject variability (i.e., S07, S11, S19, and S22) were found to possess high intrasubject variability for *cutC* gene levels determined in qPCR experiments (Figure 2).

### 3.5. Associations among Urinary TMAO, Fecal *cutC*, and Fecal Bacterial Taxa

**Figure 3:**
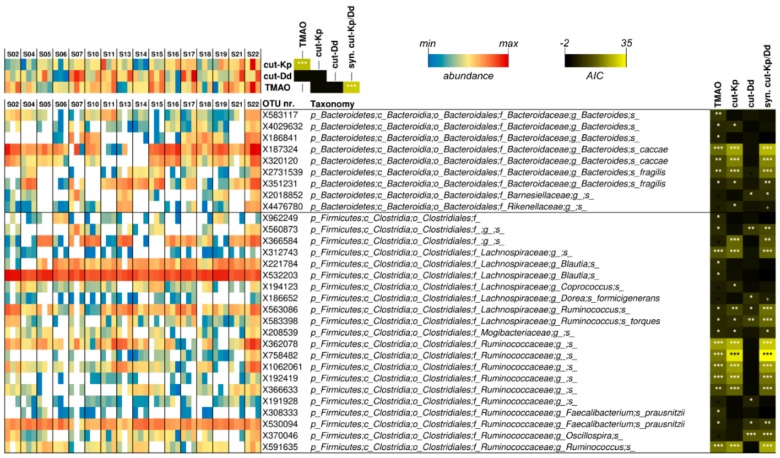
Analysis of the associations among fecal *cutC* gene abundances, fecal bacterial operational taxonomic units (OTUs), and urinary excreted TMAO carried out through a linear mixed model (LMM). Only OTUs that showed a significant association with *cutC* or TMAO are reported. The heatmap on the right represents TMAO levels, and *cutC* gene and OTU relative abundances. White boxes in the blue-yellow-red heatmap indicate that the OTU was not detected in that specific sample. The taxonomic lineage of each taxon is shown: p, phylum; c, class; o, order; f, family; g, genus; s, species. The black-yellow heatmap represents the Akaike’s information criterion (AIC) values of the LMM analysis. Asterisks indicate significant associations: * *p* < 0.05; ** *p* < 0.01; *** *p* < 0.001; ^+^, *p* < 0.1. syn. cut-Kp/Dd = synergy between cut-Kp and cut-Dd in LMM analysis.

A linear mixed model was used to infer potential significant relationships among the datasets collected from volunteers at the three time points considered (Figure 3). TMAO was significantly associated with the cut-Kp/cut-Dd synergy (*p* < 0.001). Furthermore, studying the association of the single *cut* gene types, we observed that the relationship with TMAO was mainly determined by cut-Kp (Figure 3). In addition, we found a significant association between TMAO and 23 operational taxonomic units (OTUs). Conversely, cut-Kp and cut-Dd were significantly associated with 18 and eight OTUs, respectively. Notably, most of the OTUs that were significantly associated with cut-Kp (i.e., 15 out of 18) were also associated with TMAO, confirming the relationship between these two variables. Nine of the identified OTUs belonged to the phylum *Bacteroidetes*, while the remaining 21 were ascribed to *Firmicutes*. In addition, 80% of the OTUs (n = 24) belonged to only three families: *Bacteroidaceae*, *Lachnospiraceae*, and *Ruminococcaceae*. In particular, the most significant association (i.e., *p* < 0.001) referred to *Bacteroides caccae*, an undefined *Lachnospiraceae* genus, and several undefined *Ruminoccaceae* species (for TMAO and cut-Kp), *Bacteroides fragilis*, and an undefined *Clostridiales* species (for cut-Kp only) and an *Oscillospira* species (for cut-Dd) (Figure 3).

## 4. Discussion

A growing number of studies have linked host TMAO levels to different diseases or prepathological metabolic states [28,29]. Conversely, TMAO has also been proposed as a beneficial factor that may promote protein stabilization and protect cells from osmotic and hydrostatic stresses according to a compensatory response mechanism [30]. The biological role of TMAO is therefore still debated. Nonetheless, a growing number of scientific studies have suggested that this molecule may play an important role in health and diseases [6].

It has been suggested that an important contribution to the hepatic production of TMAO is given by the TMA produced in the gut by microbial degradation of TMA-containing dietary molecules [31]. In particular, TMAO levels and their physiological consequences were shown to be significantly affected by the TMA derived from choline [15]. In this context, we studied the levels of TMAO excreted daily with urine, the composition of the intestinal microbiota, and the abundance of the choline TMA-lyase gene *cutC* in a group of healthy adult subjects with an Italian dietary pattern. The aim of this observational study was to verify whether TMAO levels excreted with the urine might be associated with the relative abundance of specific bacterial taxa and the bacterial gene *cutC* in feces. Literature focusing on the relationship among these three elements, particularly in non-diseased populations, is limited and partially contradicting [15,17,18,19].

The gene *cutC*, encoding the lyase enzyme essential for the conversion of choline into TMA [32], is not evenly distributed across bacterial taxa due to gene loss and horizontal gene transfer events that differently involve strains within the same species [15,18,33]. Therefore, predicting the choline degradation potential of a microbial ecosystem solely based on the taxonomic composition has many intrinsic limitations. The use of primers selectively targeting a specific enzymatic conserved domain may overcome this problem, permitting the selective quantification of the abundance of a gene coding for a specific enzymatic activity in the metagenomic DNA. A similar approach was used by Martinez-Del Campo et al., who designed degenerate primers for the PCR amplification of the *cutC* gene from fecal metagenomic DNA and single strains [18]. The use of degenerate primers was necessitated by the fact that the CutC protein possesses sequence heterogeneity. In particular, Martinez-Del Campo et al. showed that the amino acid sequences deduced from the predicted bacterial *cutC* genes can be clustered into two groups (clades 1 and 2, [18]), which correspond to the CutC types I and II identified by Jameson et al. within a neighbor-joining phylogenetic tree constructed from amino acid sequences of glycyl radical enzymes [32]. The same result was found in our study by generating a distance tree based on the nucleotide sequences of putative *cutC* genes (Supplementary Figure S1). In particular, cluster cut-Dd corresponded to clade 1 and CutC type I, whereas cut-Kp included sequences coding for putative proteins found in clade 2 and CutC type II reported by the authors of [18] and [32], respectively.

For this reason, we developed two nondegenerate primer pairs located at the level of the catalytic site of the encoded enzyme that were useful for the amplification in (q)PCR experiments of the two clusters of the gene *cutC*.

When the two primer sets were used with the DNA of single strains, the only positive amplification signals were obtained with the bacteria that demonstrated the ability to metabolize choline in the biotransformation assay and produce TMA, confirming the suitability of these molecular probes to target choline-TMA-converting bacteria. Specifically, the bacterial strains identified here as able to degrade choline to TMA include species previously confirmed to exert this conversion, such as *Streptococcus dysgalactiae* [18]. In addition, we found *Klebsiella oxytoca*, which was reported to harbor a putative cut gene cluster [34], but has never been confirmed phenotypically. We also identified two positive *Enterococcus* strains. Reportedly, TMA production from choline has also been described for some enterococci, but not for the species *E. gilvus*, which is often isolated from food matrices, including meat, milk, and cheeses [35,36], and for the zoonotic pathogen *E. hirae* [37].

The qPCR experiments conducted showed that putative bacterial *cutC* genes were present in the fecal samples of all healthy adult subjects investigated. The high prevalence of this bacterial gene in the human gut microbiome was reported in a previous study, in which the presence of *cutC* homologs was observed in 96.6% of the assembled stool metagenomes of healthy individuals from the Human Microbiome Project (HMP) [18].

Reportedly, most of the TMA produced in the gut is absorbed into the portal circulation by passive diffusion [38]. Then, approximately 95% of the absorbed TMA is oxidized in the liver by flavin monooxygenases and excreted in the urine within 24 h [31,39]. Therefore, in this study, we performed a quantification of TMAO levels in urine samples obtained by 24-h collection.

The data presented here revealed a marked variability of both *cutC* and TMAO levels over the three time points considered in approximately 40% of volunteers. This instability was plausibly due to the variability of the daily food consumption of each subject. In this study, volunteers were free to follow their usual diet. Therefore, the analysis of multiple time points at approximately one-week intervals was useful to address the observed temporal instability of these parameters. To the best of our knowledge, this is the first work to report the stability of intestinal *cutC* and urinary TMAO levels over time.

This study has several limitations:

1. First, we quantified the abundance of a gene of the intestinal microbiome without considering if and how much this gene was expressed. This could therefore limit the possibility of associating the abundance of this gene with its product.
2. Furthermore, the production of TMA, in addition to the presence of the bacterial gene that allows its production (*cutC*), depends on the availability of the choline substrate, which mainly comes from the diet.
3. Nonetheless, the contribution to the TMA produced in the intestine and, consequently, to the TMAO generated in the liver, derives from different chemical moieties (mainly choline, betaine, and carnitine) and includes different microbial metabolic pathways, such as those involving the carnitine monooxygenase CntAB and the glycine betaine reductase GrdH, in addition to the choline TMA-lyase CutC [40].
4. In addition, TMAO urinary levels may also depend on host factors that may largely vary from subject to subject, such as (i) the gut-to-blood barrier permeability to TMA [41], (ii) the oxidation of TMA in the liver by flavin monooxygenase [5], and the kidney function [42].
5. Finally, TMAO can also be ingested directly from foods such as fish and seafood, which are naturally rich in this molecule [43].

However, despite the limitations described above, this study showed that changes in urine TMAO levels are associated with changes in the fecal abundance of the *cutC* gene and variations in the relative abundance of several bacterial taxonomic units of the fecal microbiota. In particular, TMAO was significantly associated with the levels of a specific subcategory of the *cutC* gene, which we named cut-Kp here. This result could be explained by the relative abundance of cut-Kp, which, by qPCR results, was approximately six-times higher than that of cut-Dd. According to correlation analysis, the most important contribution to cut-Kp gene abundance is provided by *Proteobacteria*, particularly by *Enterobacteriaceae*. This result is supported by the fact that cut-Kp has been quantified with primers designed on a cluster of gene sequences having the *cutC* of the *Enterobacteriaceae* species *K. pneumoniae* as a reference. Reportedly, the analysis of human gut metagenomes revealed a high proportion of the genera *Klebsiella* and *Escherichia*, which harbor three potential TMA-producing pathways, suggesting the importance of these bacteria for TMA cycling in the human gut [44].

Most OTUs that were found to be significantly associated with TMAO also had cut-Kp, confirming the relationship between TMAO and cut-Kp levels. A few OTUs were also associated with cut-Dd. All the taxonomic units associated with TMAO and *cutC* belong to only two taxonomic orders, *Bacteroidales* and *Clostridiales*. In particular, almost all the OTUs are attributable to only three families: *Bacteroidaceae*, *Lachnospiraceae*, and *Ruminococcaceae*. Notably, these families have been identified as the most metabolically active bacteria of the human microbiota and play a dominant role in the colonic fermentation of dietary fibers [45,46]. Reportedly, many of these bacteria do not display choline-utilization activities (e.g., cut genes have never been identified in *Bacteroidetes* and *Faecalibacterium*). Nonetheless, we can hypothesize an indirect association of these bacteria with *cutC* and TMAO based on the speculation that the higher presence of these bacteria might determine a greater utilization of the available nutritional sources in the colon, reducing substrates for the remaining bacterial communities. The latter may then receive selective pressure for the expansion of the activities related to the metabolization of the residual energy and carbon sources such as choline, resulting in increased TMA production.

## 5. Conclusions

Here, we described the results of a preliminary method-development study, which suggests the existence of a relationship between the levels of TMAO excreted in urine, some intestinal taxonomic groups belonging to the most active bacterial families of the colonic microbiota, and a subgroup of the *cutC* gene ascribable to the choline-TMA conversion enzymes of *Enterobacteriaceae*, named cut-Kp, whose relative abundance can be determined with the qPCR protocol developed in this study. Nonetheless, considering the limitations listed above, particularly concerning dietary intake, it is plausible to hypothesize that the results of this study may vary in other populations.

## Supplementary Materials

The following are available online at https://www.mdpi.com/2072-6643/12/1/62/s1, Figure S1: UPGMA hierarchical clustering based on ClustalW alignment of amino acid sequences of the choline trimethylamine lyase CutC. Figure S2. Verification of choline utilization and TMA production by single bacterial strains. Figure S3: Bacterial community structure of fecal samples. Figure S4: Tukey box and whisker plots representing the most abundant genera (A) and families (B) detected by 16S rRNA gene profiling in fecal samples collected from the adult volunteers participating in this study. Figure S5: Correlations among the fecal relative abundances of the choline TMA-lyase gene *cutC* and bacterial taxa. Table S1: Bacterial strains used for the screening of choline utilization activity. Table S2: Basic characteristics of the study participants.
