# Supplementary material for: Urinary TMAO Levels Are Associated with the Taxonomic Composition of the Gut Microbiota and with the Choline TMA-Lyase Gene (cutC) Harbored by Enterobacteriaceae
Source: Nutrients. 2019 Dec 25;12(1):62. doi: 10.3390/nu12010062 (PMC7019844; doi:10.3390/nu12010062)
Supplement: Supplementary file 1 [file nutrients-12-00062-s001.pdf]

Table S1. Bacterial strains used for the screening of the choline utilization activity. All strains were available at the culture collection of the division of Food Microbiology and Bioprocesses, Department of Food, Environmental and Nutritional Sciences (DeFENS), University of Milan. Bacterial strains that displayed the choline-utilization activity are reported in red. MRS, DeMan-Rogosa-Sharpe broth; RCM, Reinforced Clostridial Medium; LB, Luria Bertani broth; cMRS, MRS supplemented with 0.05 % cysteine-HCl. MRS, M17 and reagents for LB were from Difco (Difco Laboratories Inc., Detroit, MI); RCM was from Oxoid (Basingstoke, UK).

Phylum		Bacterial strain	Growth medium	Incubation temperature
Firmicutes	1	*Carnobacterium divergens* 3b-5ba	MRS	37°C
	2	*Carnobacterium divergens* ML1-94		
	3	*Carnobacterium divergens* N14		
	4	*Carnobacterium divergens* N20		
	5	*Carnobacterium divergens* Ovb-3		
	6	*Carnobacterium maltaromaticum* F29-1		
	7	*Carnobacterium maltaromaticum* F46-1		
	8	*Carnobacterium maltaromaticum* FM-C4		
	9	*Carnobacterium maltaromaticum* ML1-95		
	10	*Carnobacterium maltaromaticum* ML1-97		
	11	*Carnobacterium maltaromaticum* N1		
	12	*Lactobacillus harbinensis* 95		
	13	*Lactobacillus helveticus* 103		
	14	*Lactobacillus acidophilus* LA5		
	15	*Lactobacillus acidophilus* NCFM		
	16	*Lactobacillus brevis* 92		
	17	*Lactobacillus casei* LMG		
	18	*Lactobacillus coryniformis* 94		
	19	*Lactobacillus delbrueckii* subsp. *bulgaricus* MIM-Y		
	20	*Lactobacillus delbrueckii* subsp. *lactis* MIM-F		
	21	*Lactobacillus fermentum* 2		
	22	*Lactobacillus helveticus* MIMLh5		
	23	*Lactobacillus johnsonii* DSM 10533		
	24	*Lactobacillus parabuchneri* 58		
	25	*Lactobacillus paracasei* 134		
	26	*Lactobacillus paracasei* DG		
	27	*Lactobacillus paracasei* S01		
	28	*Lactobacillus paracasei* Shirota		
	29	*Lactobacillus plantarum* 93		
	30	*Lactobacillus reuteri* DSM 17938		
	31	*Lactobacillus rhamnosus* 13		
	32	*Lactobacillus rhamnosus* GG		
Firmicutes	33	*Lactococcus garvieae* MIMGr	M17	37°C
	34	***Enterococcus gilvus* MD179**		
	35	***Enterococcus hirae* MD160**		
	36	*Leuconostoc mesenteroides* To 3.4		
	37	*Streptococcus agalactiae* A1.9		
	38	***Streptococcus dysgalactiae* 485**		
	39	***Streptococcus dysgalactiae* 486**		
	40	***Streptococcus dysgalactiae* A1.3**		
	41	*Weissella cibaria* CR23		
	42	*Weissella confusa* CR55		
	43	*Clostridium butyricum* DSM 10702	RCM	
	44	*Clostridium tyrobutyricum* DSM 2637		
Actinobacteria	45	*Bifidobacterium animalis* subsp. *lactis* BB12	cMRS	
	46	*Bifidobacterium bifidum* MIMBb23sg		
Proteobacteria	47	*Escherichia coli* 1.1	LB	
	48	*Escherichia coli* 1.2		
	49	*Escherichia coli* 1.3		
	50	*Escherichia coli* 2.1		
	51	*Escherichia coli* 2.2		
	52	*Escherichia coli* 2.2		
	53	*Escherichia coli* 3.1		
	54	*Escherichia coli* DSM 1003		
	55	*Escherichia coli* DSM 682		
	56	*Enterobacter agglomerans* 1.1	M17	30°C
	57	*Enterobacter agglomerans* 1.2		
	58	*Enterobacter agglomerans* 1.4		
	59	*Enterobacter agglomerans* 1.6		
	60	*Enterobacter cloacae* 1.1		
	61	***Klebsiella oxytoca* MIMgr**		
	62	***Klebsiella* sp. A1.2**		
	63	*Serratia marcescens* 1.2		
	64	*Serratia marcescens* 1.3		

Table S2. Basic characteristics of study participants.

**Subject** (n=16)	**Sex** (4F/12M)	**Age** (21-45)
S02	M	33
S04	F	34
S05	M	25
S06	M	28
S07	M	27
S10	M	33
S11	M	40
S13	F	26
S14	M	26
S15	M	45
S16	F	33
S17	M	30
S18	M	24
S19	F	28
S21	M	21
S22	M	24

Figure S1. UPGMA hierarchical clustering based on ClustalW alignment of amino acid sequences of the choline trimethylamine lyase CutC. Sequences have been selected as described in material and methods. The GenBank accession number of the nucleotide sequence corresponding to each item is reported on the right of the tree. +, *cutC* gene of *Klebsiella pneumoniae* Amm1 (Kalnins et al 2015). ++, *cutC* gene of *Desulfovibrio desulfuricans* ATCC 27774 (Craciun and Balskus 2012). *Forward* and *Reverse* refer to the primer pairs designed to amplify all sequences in the corresponding cluster.

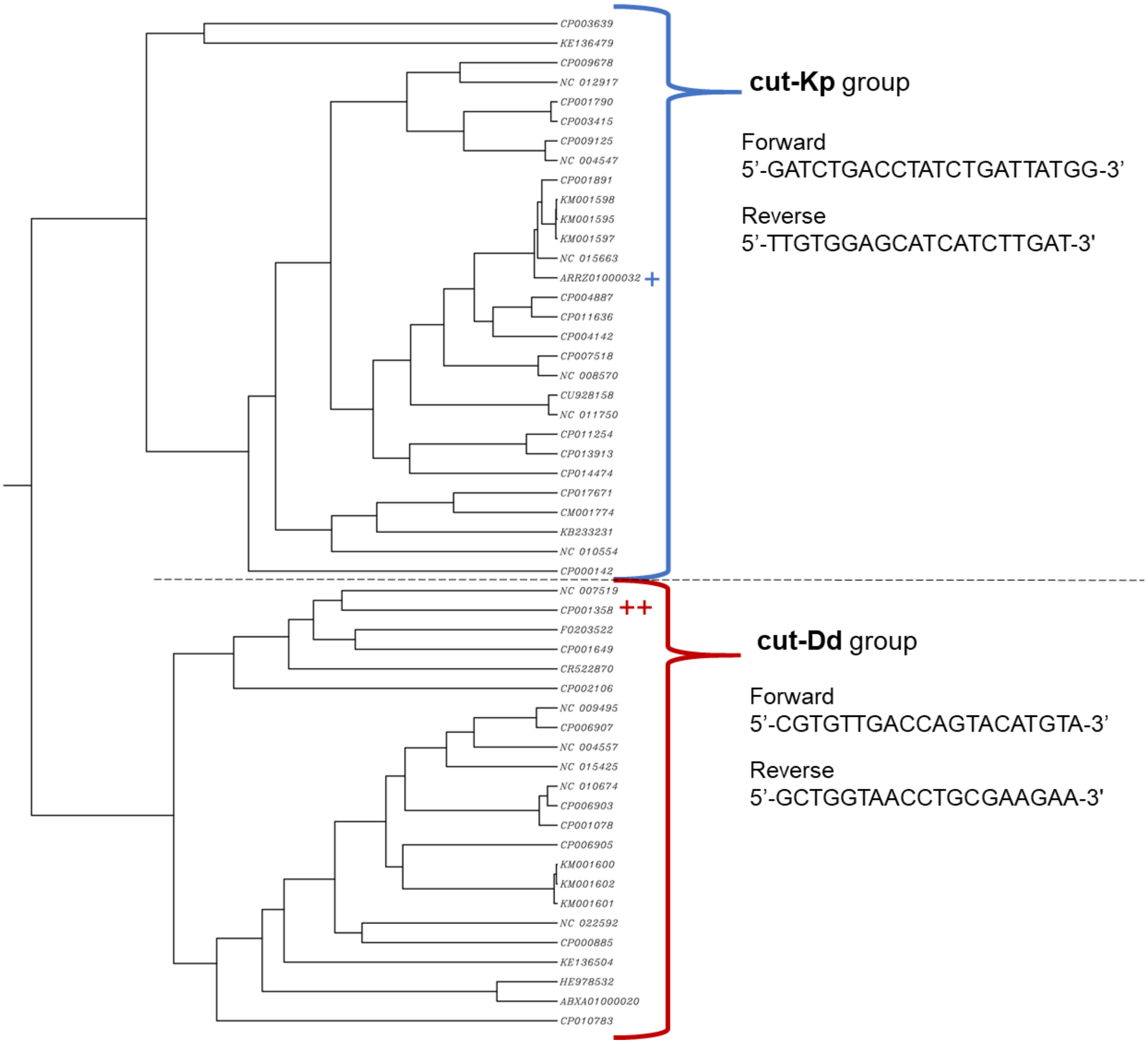

Figure S2. Verification of choline utilization and TMA production by single bacterial strains. Typical results by negative (A) and positive (B) representative strains are shown. Nuclear magnetic resonance (^{1}H-NMR) and mass spectrometry (MS) spectra are reported on the left and right, respectively. A, M17 broth supplemented with choline after incubation with *Lactococcus garvieae* MIMGr A; B, M17 broth supplemented with choline after incubation with *Klebsiella oxytoca* MIMgr; C, choline and TMA standards (50 mM in 0.1 M phosphate buffer, H_2O, pH = 6.7).

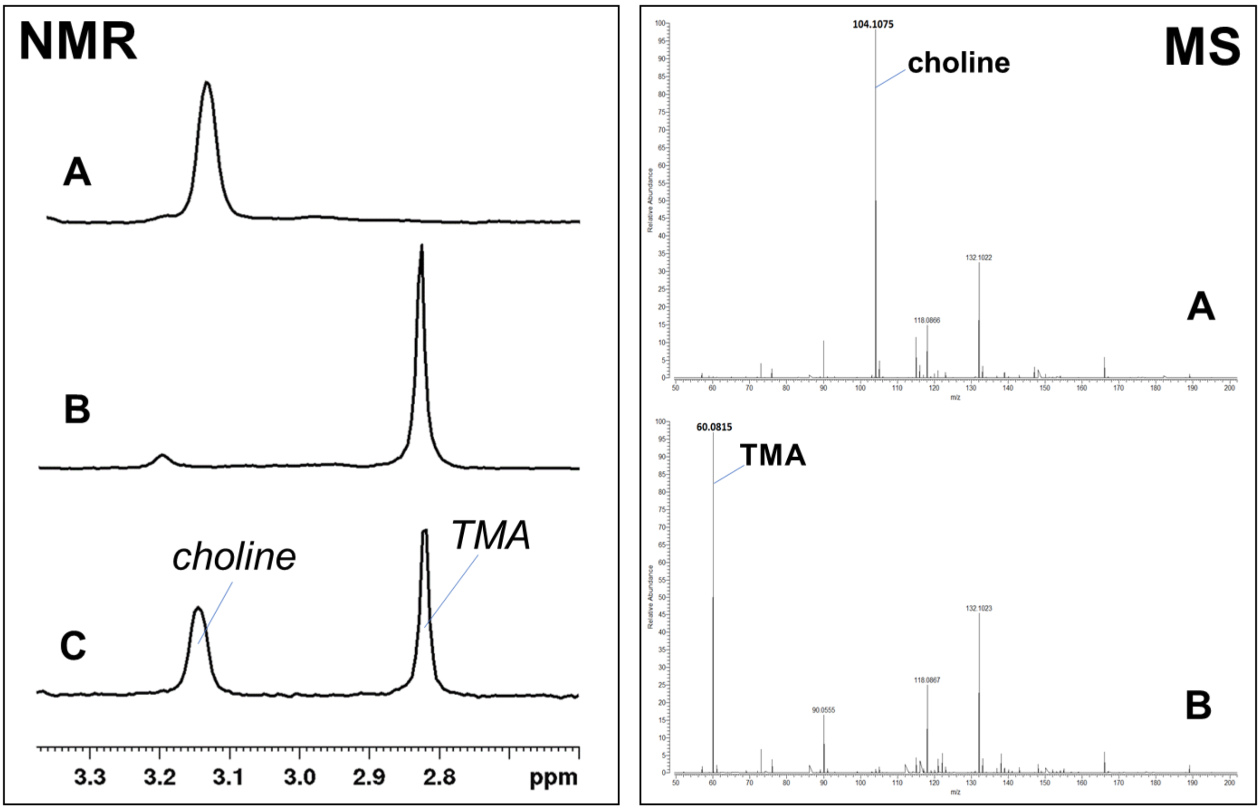

Figure S3. Bacterial community structure of fecal samples. **A**, principal coordinates analysis of weighted Unifrac distances based on 16S rRNA gene profiling data; lines connect samples from the same subject; the percentage of variance of the coordinates are explained in brackets. **B**, stacked histograms of bacterial genera in each fecal sample. The 14 most abundant bacterial genera are shown; other genera are shown in greyscale color. *und.*, undefined.

A

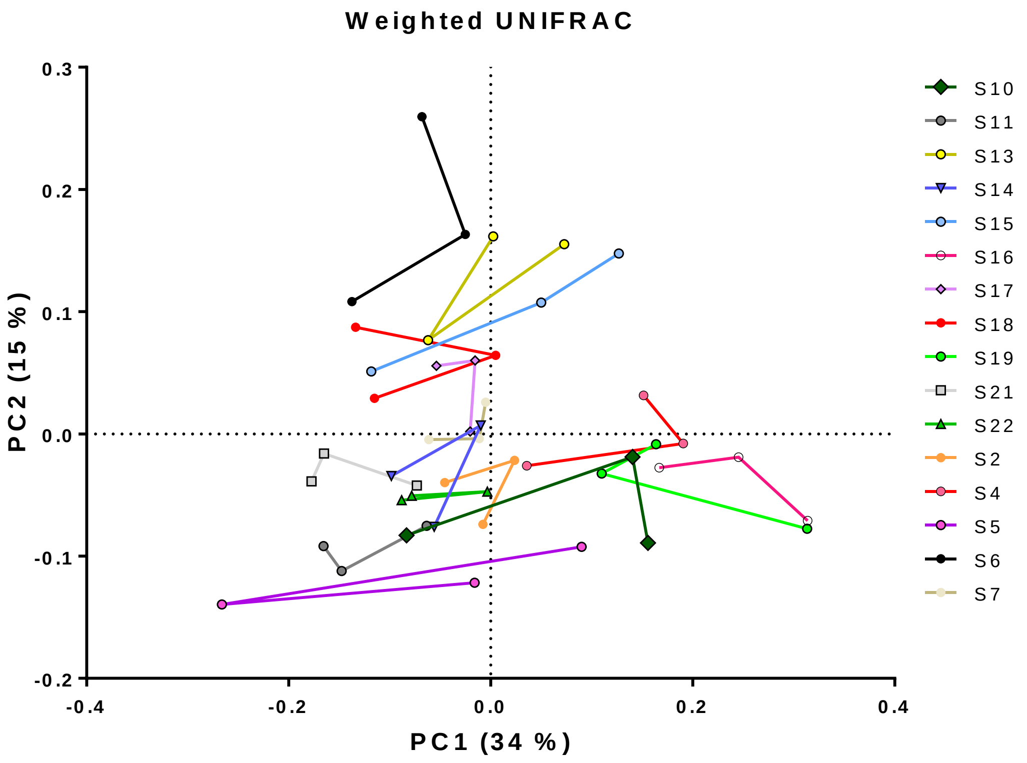

B

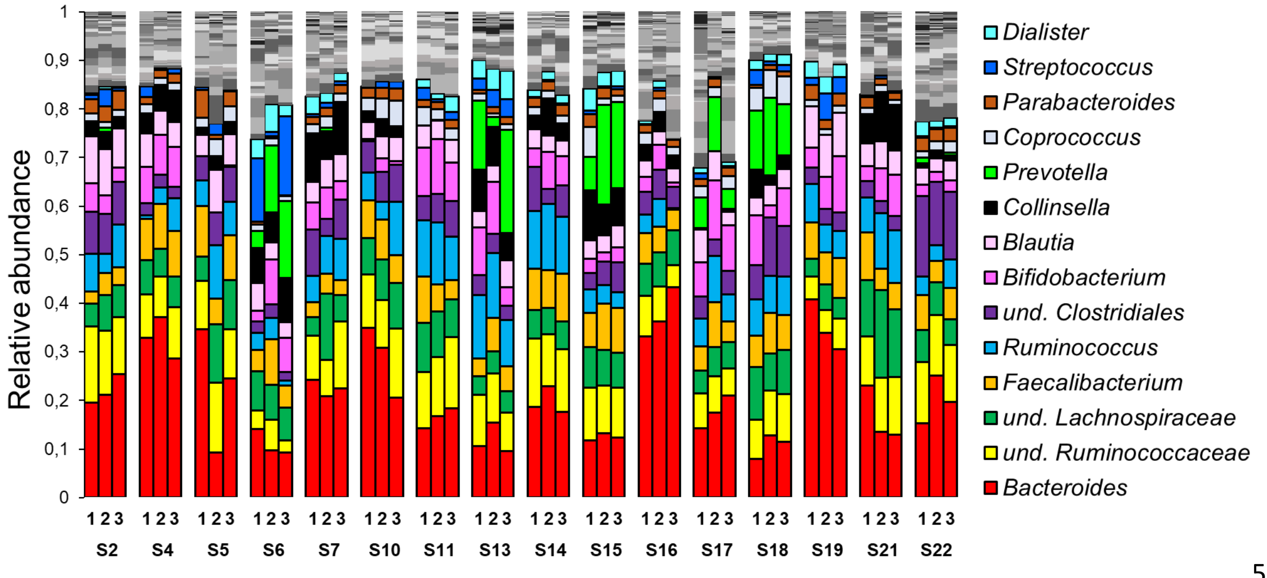

Figure S4. Tukey box and whiskers plots representing the most abundant genera (**A**) and families (**B**) detected by 16S rRNA gene profiling in fecal samples collected from the adult volunteers participating to this study.

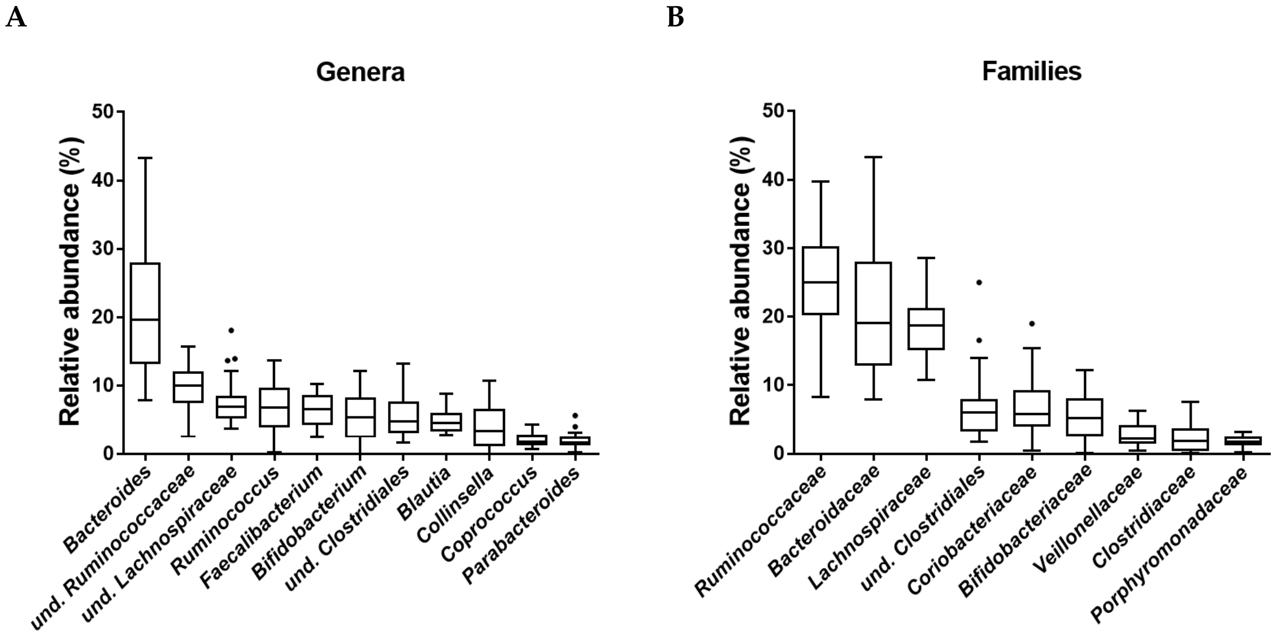

Figure S5. Correlations among the fecal relative abundances of choline TMA-lyase gene *cutC* and bacterial taxa. *cutC* abundances were determined by qPCR with primer pairs cut-Dd and cut-Kp; 16S rRNA gene profiling data were used to determine the relative abundance of bacterial taxa at the taxonomic levels of phylum (*p_*), class (*c_*), order (*o_*), family (*f_*) and genus (*g_*). The analysis was performed using median data of three measurements per subject. The heatmap represents the R value of Spearman's correlation (minimum to maximum values are indicated in heatmap legend). Asterisks indicate the Kendall rank correlation: *P < 0.05; **P < 0.01; ***P < 0.001.

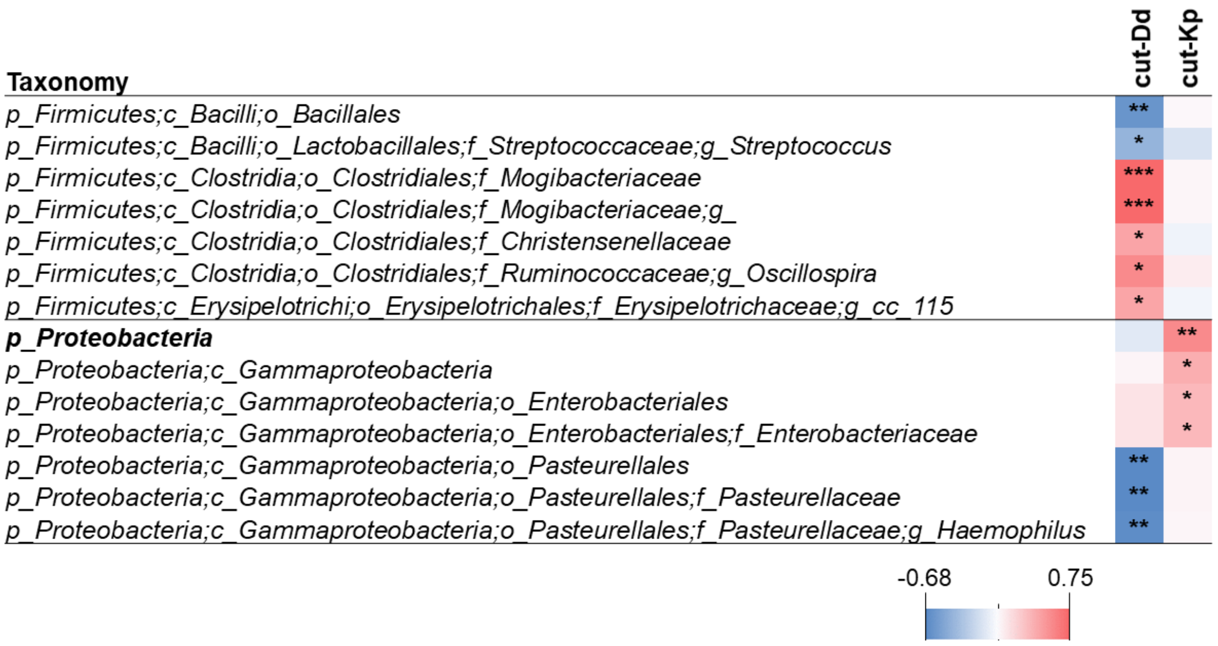

References

Craciun S, Balskus EP (2012). Microbial conversion of choline to trimethylamine requires a glycyl radical enzyme. *Proc Natl Acad Sci U S A* **109:** 21307-21312.

Kalnins G, Kuka J, Grinberga S, Makrecka-Kuka M, Liepinsh E, Dambrova M *et al* (2015). Structure and Function of CutC Choline Lyase from Human Microbiota Bacterium Klebsiella pneumoniae. *The Journal of biological chemistry* **290:** 21732-21740.
